# Up-Regulation of Hepatoma-Derived Growth Factor Facilities Tumor Progression in Malignant Melanoma

**DOI:** 10.1371/journal.pone.0059345

**Published:** 2013-03-25

**Authors:** Han-En Tsai, Jian-Ching Wu, Mei-Lang Kung, Li-Feng Liu, Lai-Hsin Kuo, Hsiao-Mei Kuo, San-Cher Chen, Elsa C. Chan, Chieh-Shan Wu, Ming-Hong Tai, Guei-Sheung Liu

**Affiliations:** 1 Institute of Biomedical Science, National Sun Yat-sen University, Kaohsiung, Taiwan; 2 Doctoral Degree Program in Marine Biotechnology, National Sun Yat-sen University, Kaohsiung, Taiwan; 3 Department of Chemistry and Center for Nanoscience and Nanotechnology, National Sun Yat-Sen University, Kaohsiung, Taiwan; 4 Department of Biological Science and Technology, I-Shou University, Kaohsiung, Taiwan; 5 Department of Internal Medicine, Chang Gung Memorial Hospital - Kaohsiung Medical Center, Chang Gung University College of Medicine, Kaohsiung, Taiwan; 6 Center for Neuroscience, National Sun Yat-sen University, Kaohsiung, Taiwan; 7 Division of Dermatology, Kaohsiung Veterans General Hospital, Kaohsiung, Taiwan; 8 Centre for Eye Research Australia, University of Melbourne, Victoria, Australia; 9 Department of Ophthalmology, University of Melbourne, Victoria, Australia; IDI, Istituto Dermopatico dell’Immacolata, Italy

## Abstract

Cutaneous malignant melanoma is the fastest increasing malignancy in humans. Hepatoma-derived growth factor (HDGF) is a novel growth factor identified from human hepatoma cell line. HDGF overexpression is correlated with poor prognosis in various types of cancer including melanoma. However, the underlying mechanism of HDGF overexpression in developing melanoma remains unclear. In this study, human melanoma cell lines (A375, A2058, MEL-RM and MM200) showed higher levels of HDGF gene expression, whereas human epidermal melanocytes (HEMn) expressed less. Exogenous application of HDGF stimulated colony formation and invasion of human melanoma cells. Moreover, HDGF overexpression stimulated the degree of invasion and colony formation of B16–F10 melanoma cells whereas HDGF knockdown exerted opposite effects *in vitro*. To evaluate the effects of HDGF on tumour growth and metastasis *in vivo,* syngeneic mouse melanoma and metastatic melanoma models were performed by manipulating the gene expression of HDGF in melanoma cells. It was found that mice injected with HDGF-overexpressing melanoma cells had greater tumour growth and higher metastatic capability. In contrast, mice implanted with HDGF-depleted melanoma cells exhibited reduced tumor burden and lung metastasis. Histological analysis of excised tumors revealed higher degree of cell proliferation and neovascularization in HDGF-overexpressing melanoma. The present study provides evidence that HDGF promotes tumor progression of melanoma and targeting HDGF may constitute a novel strategy for the treatment of melanoma.

## Introduction

Melanoma is a malignant tumor derived from melanin-producing melanocytes. The incidence of malignant melanoma has increased dramatically in recent years [1], [2]. Melanoma at the vertical tumor growth phase tends to be highly metastatic due to penetration of tumor cell to the surrounding tissues and intra-vasion into blood or lymphatic vessels, thereby increases the mortality rate [3]. Both environmental factors and genetic predisposition are important to tumor development and progression. If melanoma is not diagnosed and treated early, it will be very aggressive and become unresponsive to current therapeutic approaches. Tumor metastasis is one of the major obstacles for achieving successful clinical chemotherapy, surgery and radiotherapy for the treatment of melanoma. Therefore novel therapeutic strategies are needed to overcome tumor metastasis and to improve the survival and prognosis of melanoma patients [4].

Hepatoma-derived growth factor (HDGF) is an acidic heparin-binding protein originally isolated from the conditional medium of human hepatoma cells [5], [6]. HDGF could stimulate the proliferation of fibroblast [7], vascular smooth muscle cells [8], endothelial cells [7] and a variety of cancer cell lines including hepatoma [9], melanoma [10], lung cancer [11] gastrointestinal stromal tumors [12], pancreatic cancer [13], and gastric carcinoma [14]. Increased HDGF expression appears to correlate with the proliferating states of several cancer types hence it may be used as a novel prognostic factor for melanoma, gastrointestinal stromal tumors, esophageal carcinoma [15], pancreatic cancer, lung cancer and gastric carcinoma. Despite cumulative data indicates that HDGF is oncogenic, little is known about the underlying mechanism and signaling pathways of HDGF during carcinogenesis. Previous studies indicated that HDGF induces the transformation of NIH3T3 cells [16] and promotes cell migration [17]. Furthermore, up-regulation of HDGF has been shown in a human melanoma cell line and clinical specimen of melanoma [10]. Based on these findings, we set out to investigate the influences of HDGF expression on tumor progression of melanoma cells *in vitro* and *in vivo*. Our study showed that HDGF promoted melanoma progression in both human and mouse melanoma cells. Our findings indicate that targeting HDGF may have therapeutic potential for melanoma therapy.

## Materials and Methods

### Cell Culture

Mouse B16–F10, human A375 and A2058 melanoma cells were purchased from ATCC (Manassas, VA); MEL-RM and MM200 were gifts from Prof. Xu-Dong Zhang (University of Newcastle, Australia) [18] and were cultured in DMEM (Invitrogen, CA, USA) containing 10% fetal bovine serum (FBS; PAA, Austria), 2 mM glutamine, 100 mg/ml streptomycin (Invitrogen, CA, USA) and 100 U/ml penicillin at 37°C in 5% CO_2_ atmosphere. The luciferase-expressing B16–F10 melanoma cells were generated as previously described [19]. Human epidermal melanocytes, neonatal, moderately pigmented donor, (HEMn-MP) were purchased from Invitrogen (Carlsbad, CA) and cultured in Medium 254 supplemented with HMGS (Invitrogen, CA, USA) at 37°C in 5% CO_2_ atmosphere.

### Recombinant HDGF Protein, HDGF Antibody and Adenovirus Vectors

The recombinant HDGF protein (rHDGF) and anti-HDGF antibody were generated as described previously [9]. The recombinant adenoviruses containing HDGF genes (Ad-HDGF) and HDGF shRNA (Ad-HDGF shRNA) were generated as previously described [20] and were used to overexpress and silence HDGF gene expression respectively. Recombinant adenoviruses containing green fluorescent protein (Ad-GFP) were used as controls. The optimal condition for adenoviral vector transduction of human melanoma cells (A375 and A2058) and mouse melanoma cells (B16–F10) were determined to be a multiplicity of infection (MOI) of 100 and 1000 respectively, at which >80% of the cells expressed the transgene without overt cytotoxicity.

### Quantitative Real-time PCR

Quantitative PCR was performed to verify the expression of HDGF as described previously [21]. Total RNA from human melanoma cell lines (A375, A2058, MEL-RM and MM200) and human epidermal melanocytes (HEMn) cells was extracted with Trizol reagent according to manufacturer’s instructions (Ambion, CA, USA) and reverse-transcribed to cDNA using TaqMan high performance reverse transcription reagents (Applied Biosystems, VIC, Australia) at 25°C for 10 min, 37°C for 2 hrs followed by 85°C for 5 sec in a Thermal cycler (BioRad-DNA Engine). The real-time PCR reactions were performed in a 7300 system (Applied Biosystems, VIC, Australia) using TaqMan Universal PCR master mix and predesigned gene specific probes and primer sets for human HDGF (Hs00610314_m1, Applied Biosystems). Data were normalized to GAPDH (4326317E, Applied Biosystems) and expressed as fold changes over that in control treatment group.

### Western Blot Analysis

Whole cell protein extracts were prepared as described previously [20]. Proteins were separated on 8% to 16% Amersham™ ECL™ gels and western blotted with antibodies for HDGF (1∶1000 dilution), α-SMA (1∶1000 from Sigma Inc; MO), E-cadherin (1∶500 from Santa Cruz Inc; CA), vimentin (1∶500 from Santa Cruz Inc; CA) and β-actin (1∶5000 from Chemicon).

### Colonies Formation Assay

The flat colony formation assay was performed as previously described [20]. Cells seeded in DMEM supplemented with 0.5% FCS were incubated with rHDGF (10 ng/ml) for 7–10 days prior to crystal violet staining for colony counting. Cells were infected with Ad-HDGF, Ad-HDGF shRNA and Ad-GFP for 24 hrs and were then incubated in DMEM supplemented with 10% FCS for 7–10 days prior to colony counting. Cells were then fixed in 4% paraformaldehyde and incubated in crystal violet (0.01% in 10% buffered formalin; Sigma, St. Louis, MO) for 30 min for colony counting.

### Invasion Assay

Cell invasion was measured using a trans-well assay in a Boyden chamber (48-well plate), using a polycarbonate filter (8 µm pore size; Nucleopore, Costar, Cambridge, MA, USA) as previously described [20]. The polycarbonate filter was coated with Matrigel (2 mg/ml; BD Biosciences) at 4°C for 1 hr and placed on the lower compartment of a 48-well plate with DMEM containing 10% FCS as a chemoattractant. For studies using rHDGF, cells (1×10^5^ cells/ml) supplemented in DMEM containing PBS or rHDGF (10 ng/ml) were seeded in wells (50 µl/well) of the upper compartment. For adenovirus gene delivery, cells (1×10^5^ cells/ml) were seeded and harvested after infection with adenovirus vectors for 24 hrs. The cells (5×10^3^ cells/well) were then place in the wells (50 µl/well) of the upper compartment. After 24 hrs of incubation, the invading cells on the lower surface of the polycarbonate filter were stained with 0.5% crystal violet and observed by microscopy. The number of invading cells was counted from at least four high-power fields (×200 magnification) per assay, and data are presented as averages of triplicate experiments.

### Primary and Metastasis Melanoma Models

All animal experiments were carried out under protocols approved by Animal Care and Use Committee (IACUC) of National Sun Yet-Sen University (Kaohsiung, Taiwan; approval ID, NSC 98-2320-B-110-004-MY3/97014).

B16–F10 cells are infected with Ad-GFP, Ad-HDGF and Ad-HDGF shRNA at MOI 1000 for 24 hrs. Harvested, washed three times with phosphate buffered saline (PBS) in order to minimize viral contamination in C57BL/6 mice. To induce primary melanoma, B16–F10 cells were subcutaneously injected into male C57BL/6JNarl mice (4–6 weeks old; 5×10^5^ cells in 0.1 ml PBS; n = 13–15) to induce tumor growth. Subsequently, tumor volumes were measured with a dial-caliper using the formula: width^2^×length×0.52. For tumor-suppression studies, experiments were terminated when tumor burden exceeded 10% of animal’s normal body weight.

To induce metastatic melanoma, luciferase-expressing B16–F10 cells were injected into tail vein of male C57BL/6JNarl mice (4–6 weeks old; 5×10^5^ cells in 0.1 ml PBS; n = 13–15) to induce pulmonary metastasis. Metastatic progression was monitored and quantified by a non-invasive bioluminescence (described below).

### Bioluminescence Imaging

After animals were anesthetized with a cocktail of ketamine:xylazine (4∶1) in PBS, mice were injected intraperitoneally with 100 µl of D-luciferin (Promega; 20 mg/ml) and placed in the imaging chamber (IVIS Imaging System 50 Series, Caliper Life Sciences; Hopkinton, MA) on a platform warmed to 37°C. A gray scale body surface image was obtained in the chamber under dim illumination followed by 1 minute acquisition and overlay of the pseudocolor images. The spatial distribution and quantity of photon counts emitted by cells producing luciferase within the animals were represented by colored pixels produced by the computer.

### Immunohistochemical Analysis

Paraffin-embedded 5 µm-thick tumor sections were stained with hematoxylin and eosin to examine tissue morphology. For immunohistochemical studies, tissue sections were de-waxed and treated in proteinase K (Dako, Denmark) for 8 minutes. The sections were then washed in TBS (pH 7.5) and quenched with 3% H_2_O_2_ in methanol for 10 minutes to block endogenous peroxidase. After TBS washes, sections were incubated for 20 min in blocking solution (10% goat serum), followed by an overnight incubation with primary antibody, Ki-67 (rat anti-mouse undiluted; Vision BioSystem Co, Balliol, UK), for 1 hr at room temperature. Following repeated washes with PBS, horseradish peroxidase/Fab polymer conjugate (Polymer detection system, Zymed, USA) was applied to the sections and then incubated for 30 min. Sections were incubated with peroxidase substrate 3, 3′-diaminobenzidine (Dako, Denmark) for 1–2 min and counterstained with Gill’s hematoxylin for 2 sec. Negative controls were incubated with a corresponding serum isotype (rat IgG).

For quantifying the immunostaining of Ki67, each sample was randomly captured by microscopy for at least five independent fields. The number of Ki67 positive cells were counted and calculated from five independent fields with a 40× microscopic field.

### Immunofluorescence Analysis for Neovascularization

The optimum cutting temperature (Thermo Co. Pittsburgh, PA, USA)-embedded tissue blocks were sectioned into 2 µm slices and mounted on poly-L-lysine-coated slides. After antigen retrieval with microwave in 10 mM citrate buffer for 15 min, and then proteins blocked for 60 min. The mouse anti-human CD31 antibody (1∶50 dilution from Novocastra), was applied onto sections and incubated at room temperature for overnight followed by repeated washes with PBS. Then, tissue sections were incubated with fluorescein 5-isothiocyanate (FITC)-labeled secondary antibody (Jackson ImmunoResearch Inc. West Grove, PA, USA) for 30 min at room temperature. After mounting in anti-Fade media (Dako Inc; Carpinteria, CA, USA), the slides were visualized under a fluorescence microscope.

For quantifying the CD31 positive blood vessels, each sample was randomly captured by microscopy for at least five independent fields. The number of CD31 positive vessels were counted and calculated from five independent fields with a 20× microscopic field.

### Transfection Experiments and Luciferase Measurements

For transient transfections, adenovirus infected B16–F10 cells (in a six-well plate) were grown to 80% confluence and were then transfected with plasmid DNA using a lipofectamine plus method (Invitrogen) according to the manufacturer’s instructions. For promoter activity assay, cells were co-transfected with 1 µg E-cadherin-driven luciferase vector (generous gift from Dr. Yu-Sun Chang, Chang Gung University, Taiwan) [22] and the 0.2 µg Renilla reniformis luciferase reporter vector (Promega, Madison, WI). The luciferase activities in cells were determined using a Dual-Light kit (Promega, Madison, WI) in a luminometer (Microlumat Plus LB96V; Berthold Technologies, Bad Wildbad, Germany) and normalized with that of R. reniformis luciferase according to manufacturer’s instructions.

### Statistical Analysis

Differences between the groups were statistically evaluated using the t test or one-way ANOVA with post-doc analysis. The results are presented as mean ± SEM. All *P* values were two-tailed, and a *P* value of less than 0.05 was considered to be statistically significant.

## Results

### High Level of HDGF Expression in Human Melanoma Cells

**Figure 1:**
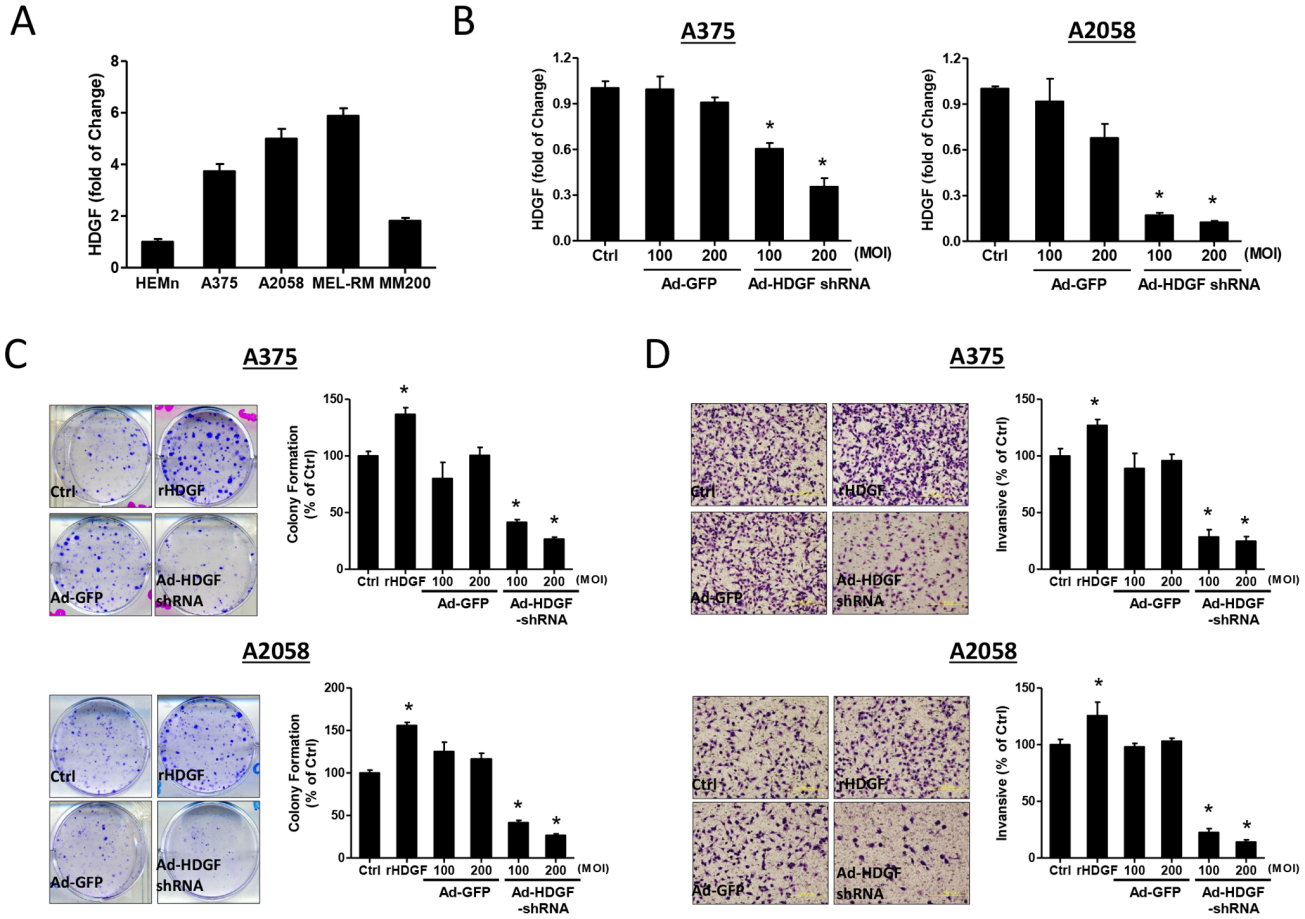
Expression level of HDGF and effects of HDGF on tumorgenicity of human melanoma cells *in vitro.* (A) The gene expression of HDGF in human epidermal melanocyte (HEMn) and melanoma cell lines (A375, A2058, MEL-RM and MM200) was measured by qRT-PCR. The relative gene expression level of HDGF was normalised to GAPDH. No statistical analysis was performed (melanocyte group is populated by only one type of cell line). (B) A375 and A2058 cells were infected with adenoviral vector at different MOI (100 or 200) then evaluated gene expression level of HDGF. (C) Representative images illustrating the effects of rHDGF (10 ng/mL) and Ad-HDGF shRNA (at 200 MOI) and Ad-GFP (at 200 MOI) on colony formation identified by crystal violet stains in A375 and A2058 cells. Quantitative measures of colony formation by counting the number of crystal violet positive cells. (D) Cells invaded through polycarbonate membrane (10 mm pore size) were stained with Giemsa. Representative photomicrographs of migrated cells through the Matrigel-coated filter were quantified in A375 and A2058 cells. All data are expressed as mean ± SEM from 3 experiments. *P<0.05 compared to control, one-way ANOVA with post-doc analysis.

The endogenous level of HDGF expression was detected using qRT-PCR analysis. As showed in Fig. 1A, human melanoma cell lines (A375, A2058, MEL-RM and MM200) had higher levels of HDGF gene expression, whereas human epidermal melanocytes (HEMn) expressed less. To investigate the correlation of HDGF expression with tumor progression, we assessed the effects of HDGF inhibition on melanoma using small interfering RNA (shRNA) in human A375 and A2058 melanoma cells. Recombinant adenovirus encoding HDGF shRNA (Ad-HDGF shRNA) was generated for gene silencing study. Following HDGF shRNA gene delivery to A375 and A2058 cells for 72 hrs, HDGF mRNA levels were significantly reduced by 60–70% as compared with controls (Fig. 1B).

### Exogenous Application of rHDGF Stimulates Invasion and Colony Formation of Melanoma Cells, whereas HDGF Knockdown Inhibits these Processes

Since tumorigenesis is involved of multiple oncogenic processes including proliferation, anchorage-independent growth and invasion, we investigated whether modulation of HDGF expression affected these processes. An application of rHDGF (10 ng/mL) for 7–10 days increased colony formation, whereas HDGF shRNA gene delivery significantly inhibited colony-forming capability in both A375 and A2058 melanoma cells (Fig. 1C). We also examined tumor cell invasion following treatment with rHDGF (10 ng/mL) or gene knockdown with HDGF shRNA gene delivery. It was found that HDGF-treated melanoma cells showed a significant elevation in the extent of cell invasion compared to untreated or Ad-GFP control groups (P<0.05; Fig. 1D). These results indicate that HDGF promotes the tumourigenic activity of melanoma cells.

### Overexpression of HDGF in Melanoma Cells Promotes Melanoma Growth in Tumor Bearing Mice

**Figure 2:**
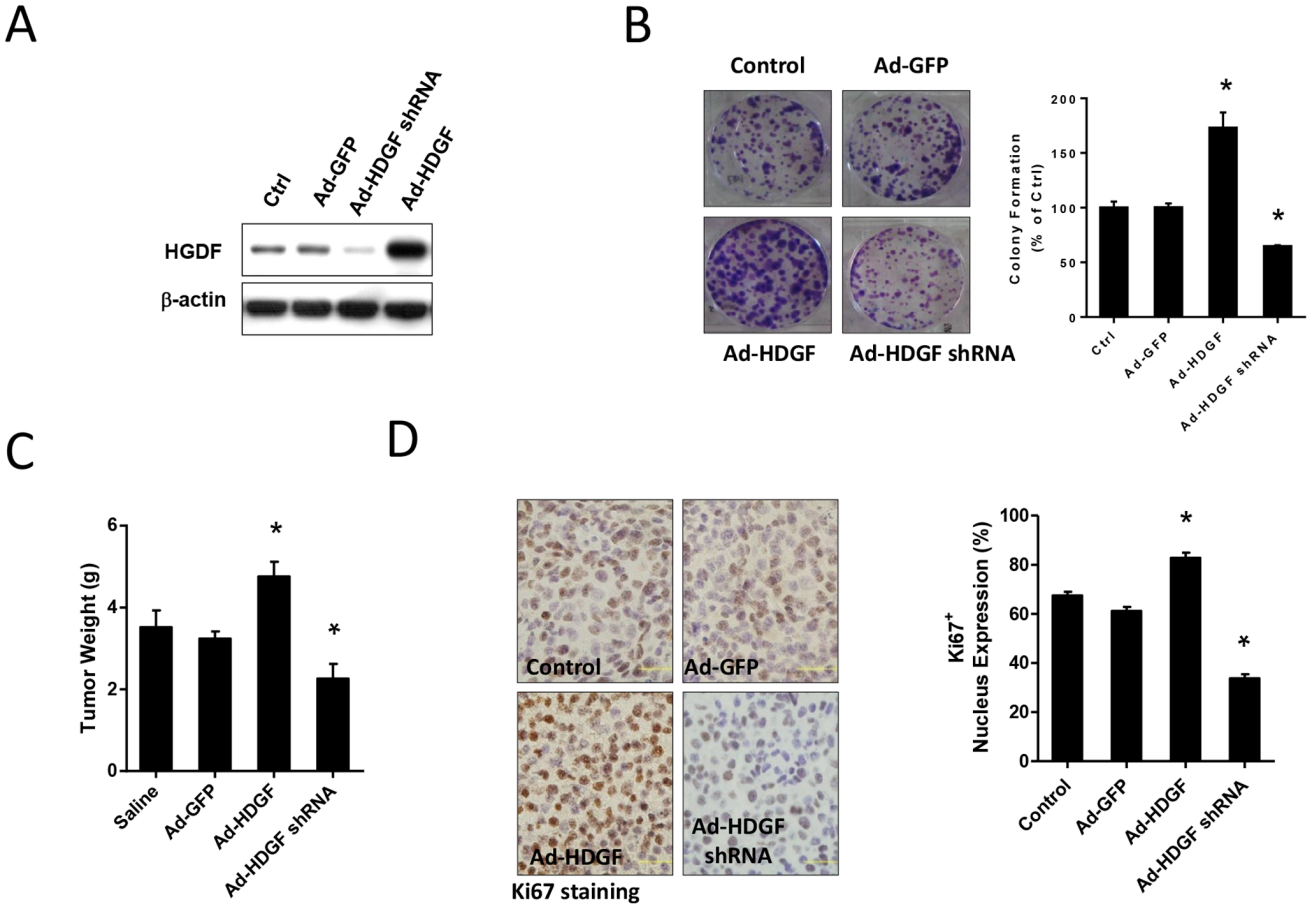
Effects of HDGF overexpression or gene silencing on tumorigenesis of melanoma cells *in vitro* and *in vivo.* (A) A representative western blot demonstrating HDGF protein expression in B16–F10 melanoma cells infected with Ad-GFP, Ad-HDGF shRNA and Ad-HDGF. The β-actin was used as controls. (B) Representative photos and quantification of colony formation ability assay (from 3 experiments). (C) Weights of tumors isolated from C57BL/6 mice. Melanoma cells infected with recombinant adenovirus for 24 hrs were subcutaneously implanted into mice over 28 days. Tumors were then excised and weighted (n = 10/each group). (D) The representative profile of Ki-67 expression in tumor sections from mice inoculated with B16–F10 melanoma cells infected with Ad-GFP, Ad-HDGF and Ad-HDGF shRNA. Bar chart shows proliferation index of Ki-67 positive cells in tumor tissues (n = 6/each group). All data are expressed as mean ± SEM. *P<0.05 compared to Ad-GFP-treated groups, one-way ANOVA with post-doc analysis.

To evaluate the effect of HDGF expression on melanoma growth *in vivo*, we employed the B16–F10 syngeneic melanoma models. We first confirmed that an infection of B16–F10 melanoma cells with shRNA (Ad-HDGF shRNA) targeting HDGF could silence HDGF expression and Ad-HDGF increased HDGF expression. Western blot analysis demonstrated that Ad-HDGF shRNA gene delivery significantly reduced protein levels of HDGF, whereas Ad-HDGF gene delivery significantly elevated HDGF expression in B16–F10 cells (Fig. 2A). We subsequently investigated the colony-forming capability of B16–F10 cells after HDGF known down or overexpression. It was found that HDGF shRNA-transduced melanoma cells significantly reduced colony formation and such responses were potentiated by HDGF overexpression (Fig. 2B), implicating a role of HDGF in anchorage-independent growth.

To further evaluate the role of HDGF in melanoma progression, mice received subcutaneous injection of infected B16–F10 cells to assess tumor growth over 28 days. It was found that tumors derived from Ad-HDGF-infected cells had the largest tumor burden (Fig. 2C). In contrast, the tumor burden of melanoma from Ad-HDGF shRNA-infected cells was significantly attenuated.

### Overexpression of HDGF Enhances Tumor Proliferation and Promotes Blood Vessels Formation in Melanoma Tissue

**Figure 3:**
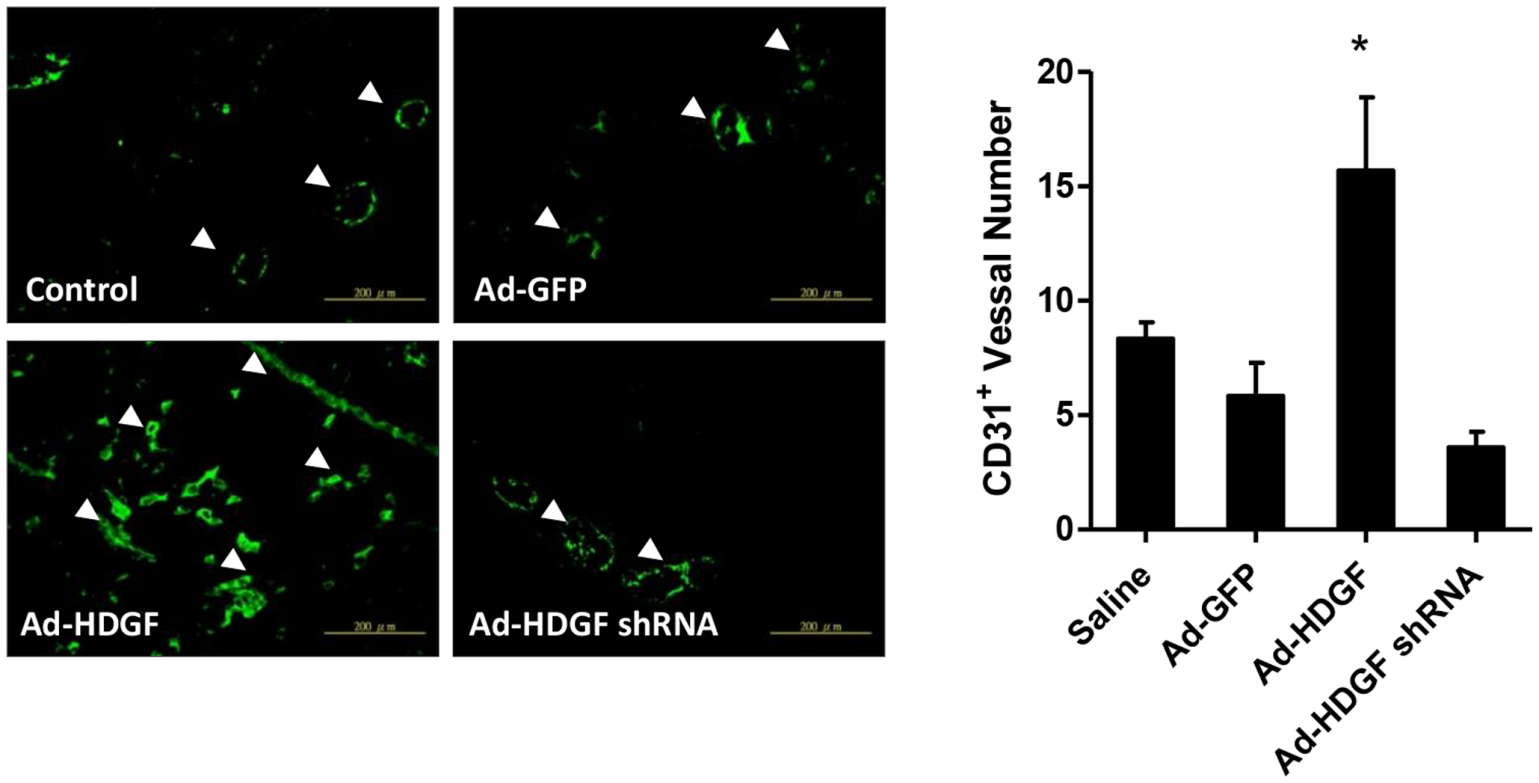
Effects of HDGF overexpression or gene silencing on tumor angiogenesis in B16–F10 melanoma tissue. After 28 days of tumor inoculation, melanoma developed in C57BL/6 mice was dissected and sectioned and subjected to immunofluorescent detection of CD31 positive blood vessels. Arrows indicate the blood vessels. Bar chart shows the degree of neovascularisation in tumor sections (n = 6). Scale bar: 200 µm. All data are expressed as mean ± SEM. *P<0.05 compared to Ad-GFP-treated groups, one-way ANOVA with post-doc analysis.

To investigate the mechanisms underlying different tumor size, the extent of tumor proliferation and neovascularization was assessed with respective Ki-67 and CD31 antibodies. A significant increase in Ki-67-positive proliferating cells was found in tumors from Ad-HDGF-infected cells. Conversely, tumors from Ad-HDGF shRNA-infected cells showed a reduction in proliferation index (Fig. 2D). Besides, the CD31-positive neovascularization was significantly elevated in tumors isolated from Ad-HDGF group (Fig. 3). Therefore, HDGF overexpression appears to create a favourable environment for tumor progression by promoting tumor proliferation and neovascularisation.

### Overexpression of HDGF Enhances Metastasis through the Regulation of Epithelial-to-Mesencyhmal Transition (EMT) Changes in Melanoma

**Figure 4:**
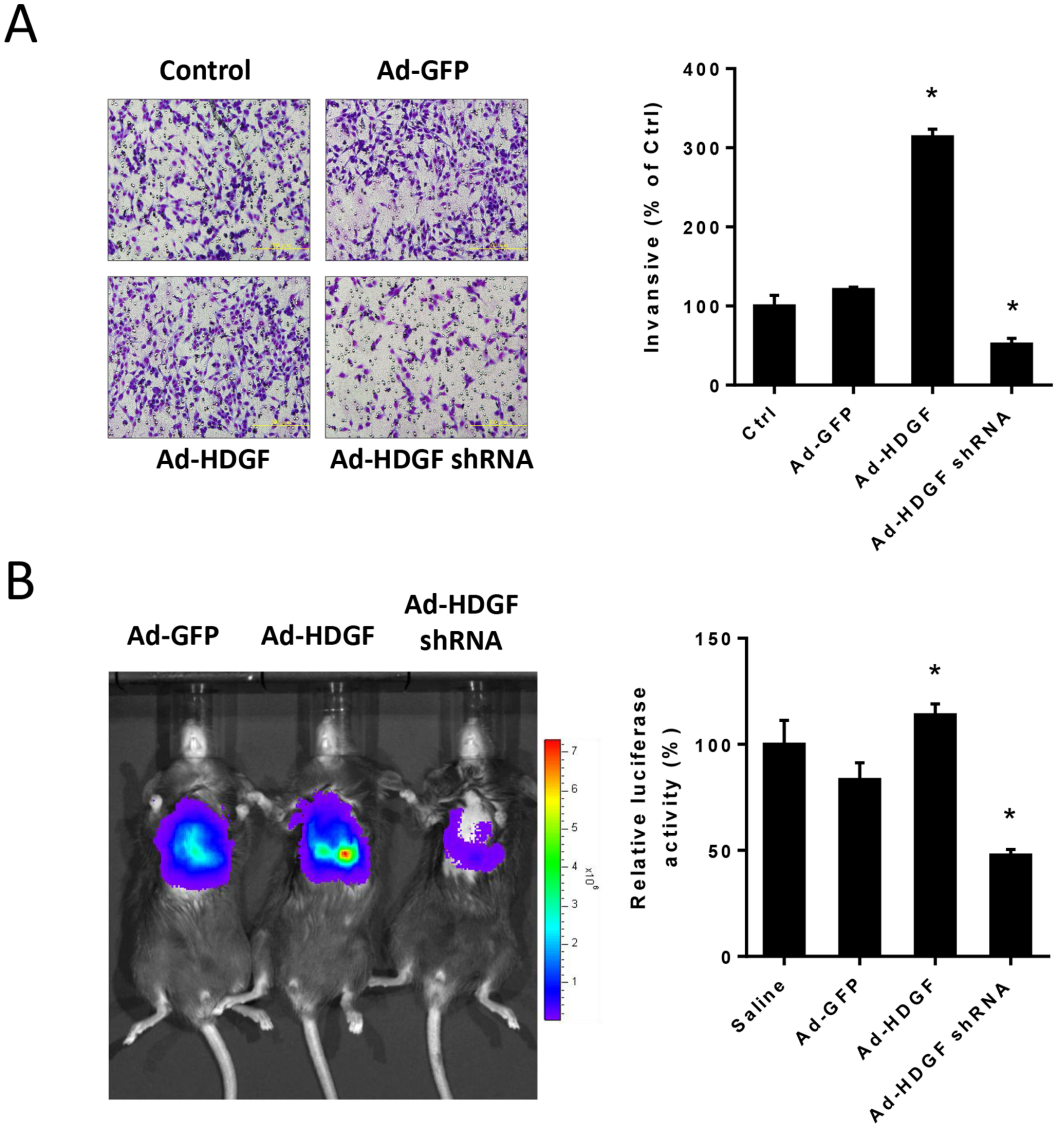
Effects of HDGF overexpression or gene silencing on metastatic capability of melanoma cells *in vitro* and *in vivo.* (A) After infection with adenovirus vectors (Ad-GFP, Ad-HDGF and Ad-HDGF shRNA), cells were subjected to cell invasion assay. Representative photomicrographs and the degree of migrated cells through Matrigel-coated filters. (B) Live images and the quantification of photon units for 14 days were shown (n = 10/each group). All data are expressed as mean ± SEM. *P<0.05 compared to Ad-GFP-treated groups, one-way ANOVA with post-doc analysis.

To evaluate whether HDGF contributes to the regulation of metastasis in melanoma, we first investigated the extent of invasion of B16–F10 melanoma cells after HDGF known down or overexpression *in vitro*. It was found that HDGF overexpression significantly enhanced the matrix-penetrating capability in B16–F10 cells, but such response was retarded by HDGF shRNA (Fig. 4A).

We then examined the efficacy of HDGF overexpression for promoting lung metastasis in mice inoculated with B16–F10 melanoma cells for 14 days. To assess the extent of metastasis, mice were administrated with B16–F10 cells tagged with firefly luciferase (Luc-B16–F10) for bioluminescence analysis. It was shown that bioluminescence intensities in lungs of mice receiving Ad-HDGF-treated cells were greater than those of control groups on day 14. In contrast, metastasis of lung melanoma from treated with Ad-HDGF shRNA was significantly attenuated (Fig. 4B).

**Figure 5:**
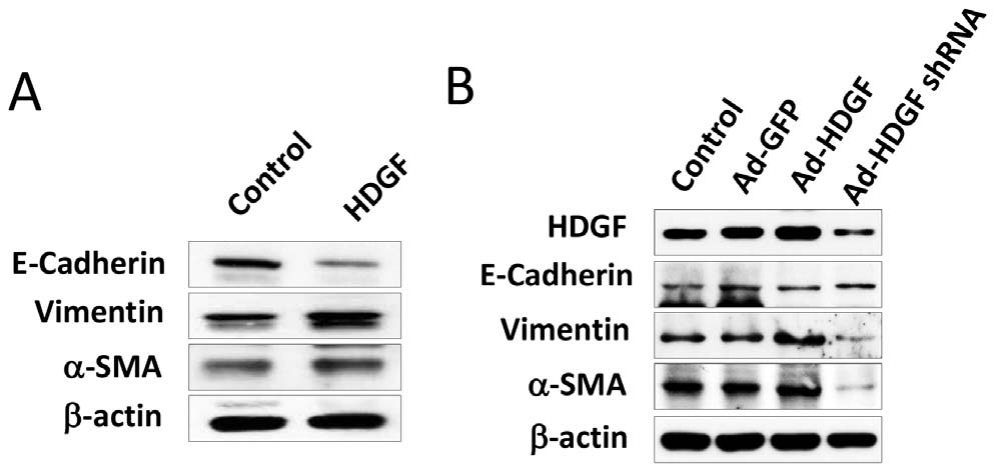
Effects of HDGF on EMT change in melanoma. (A) Effect of exogenous rHDGF (10 ng/mL) application or (B) gene delivery by Ad-GFP, Ad-HDGF and Ad-HDGF shRNA on EMT proteins: E-cadherin, vimentin and α-smooth muscle actin (SMA). EMT relative protein expression in B16–F10 cells were analysed by western blot.

Our recent study demonstrated that HDGF up-regulation is correlated with recurrence, lymph node metastasis and EMT in breast cancer patients [20]. Moreover, HDGF protein also regulates the EMT in breast cancer cells through modulation of E-cadherin and vimentin expression [20]. Thus, we evaluated whether HDGF affected the EMT of melanoma cells by examining the expression of EMT marker molecules. Western blot showed that exogenous rHDGF (10 ng/mL) treatment significantly reduced the protein level of E-cadherin, an epithelial marker, while it elevated the protein levels of mesenchymal markers, including vimentin and α-SMA in melanoma cells (Fig. 5A). E-cadherin promoter analysis also confirmed that exogenous HDGF application reduced the promoter activity of E-cadherin in B16–F10 melanoma cells (Fig. S1A). Similarly, HDGF overexpression by Ad-HDGF gene delivery reduced the protein level and promoter activity of E-cadherin, while it elevated the protein levels of vimentin and α-SMA in B16–F10 melanoma cells (Fig 5B and Fig. S1B). Conversely, Ad-HDGF shRNA infection potentiated protein level and the promoter activity of E-cadherin, and reduced the protein levels of vimentin and α-SMA in B16–F10 melanoma cells (Fig 5B and Fig. S1B). These findings suggest that HDGF promotes melanoma metastasis through EMT modulation.

## Discussion

**Figure 6:**
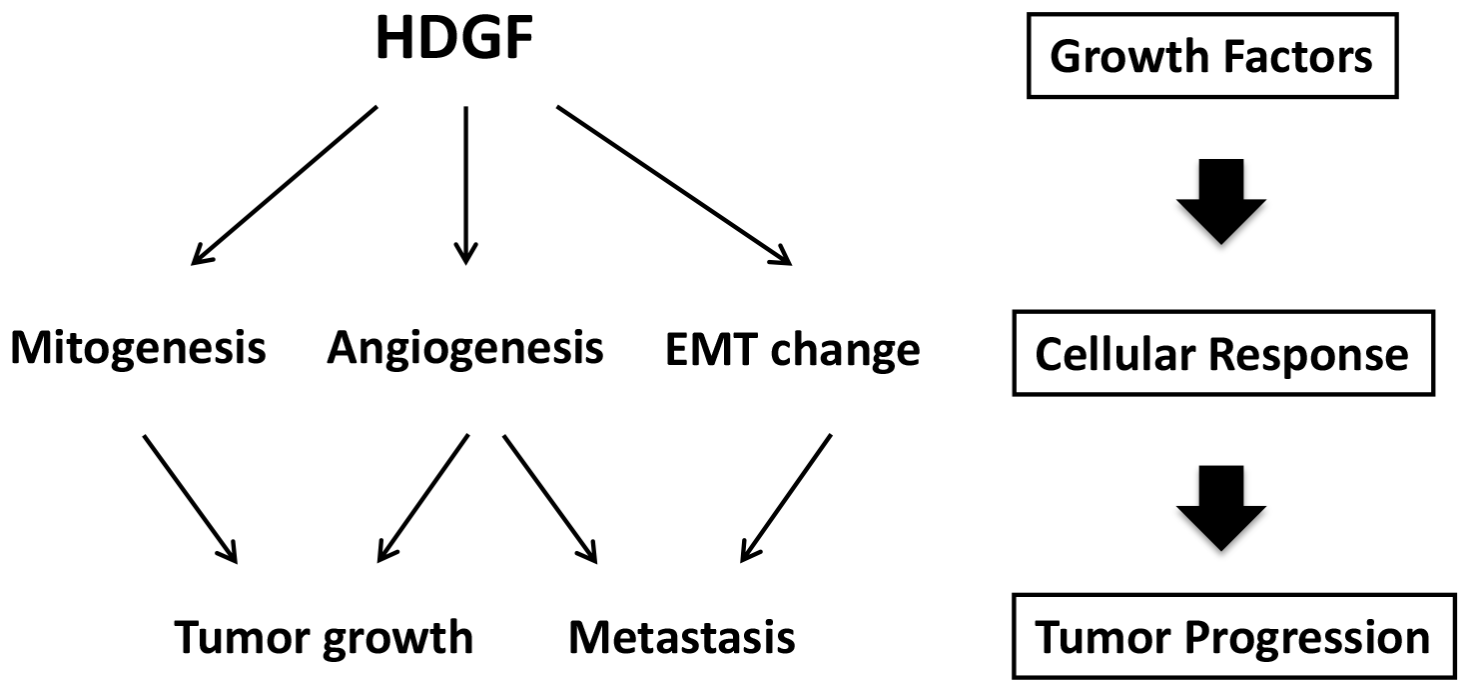
Schematic representation of HDGF stimulated tumor progression in melanoma.

Our study provides two novel findings that HDGF promotes melanoma progression in both human and mouse melanoma cells and it encourages cell invasion and metastasis in a mouse model of melanoma. Knocking down HDGF with HDGF shRNA demonstrates a significant inhibition of tumor growth and retards lung metastasis by reducing tumor invasion and colonization in established melanoma. We also show that HDGF modulates EMT changes by down-regulating E-cadherin and up-regulating vimentin and α-SMA, which contributes to tumor cell invasion and metastasis in melanoma *in vitro* and *in vivo*. Targeting HDGF may have therapeutic potential for melanoma therapy (Fig. 6).

The major risk factors for the development of melanoma have been gradually identified, and some of the processes involved in the molecular pathogenesis of melanoma have been elucidated in recent years. HDGF appears to be an important growth factor for melanoma growth and its expression could be shuttled between cytoplasm and nucleus under different conditions such as cell proliferation cycle and state of differentiation [23]. In melanoma, Bernard *et al.* revealed that HDGF was expressed in melanomas but was poorly expressed in non-tumorigenic melanocytes [10]. Moreover, the expression of HDGF correlates with tumor progression and the frequency of HDGF expression increases from benign nevi to late stages of melanoma [10]. The results from exogenous application of HDGF and genetic modulation of HDGF in the present study support such notion that HDGF elevation promotes the neoplastic transformation in melanocytes and HDGF expression regulates the melanoma progression. However, a recent study demonstrated that melanocyte-specific HDGF overexpression did not promote oncogenic transformation of melanocytes. Therefore, expression of HDGF appears to correlate with melanoma malignant behaviours such as proliferation and invasion, but not to the oncogenic transformation potentials of melanocytes. Indeed, our data showed that exogenous HDGF application stimulates the capacity of colony formation and invasion of human melanoma cells. In contrast, down-regulation of HDGF expression with Ad-HDGF shRNA led to cell growth arrest and attenuated cell invasion. In addition, by employing the syngeneic B16–F10 melanoma models, overexpression or knockdown of HDGF influenced the tumor progression such as tumor growth and metastasis in mice. These results demonstrate a positive correlation of HDGF expression with melanoma aggravation and distant metastasis, but the underlying mechanisms remains to be further determined.

Tumor neovascularization plays a critical role in tumorigenesis of many tissue types since normal vasculature does not support tumor growth. Tumor neovascularization is an essential constituent for the development, invasion and metastasis of tumour [24]. Several groups demonstrated that angiogenesis occurs in tumor development in skin carcinogenesis, suggesting that an establishment of blood vessel networks is important for tumor progression [25]. Indeed, the striking correlation between the degree of neovascularisation and metastatic melanoma nodules strongly indicates a dependency of newly colonized melanoma cells on neovascularization. Since HDGF is a well-known pro-angiogenic factor [26], HDGF may promote tumor progression through the modulation of angiogenesis in melanoma. In the present study, we demonstrated that HDGF shRNA gene delivery significantly reduced the level of blood vessel growth in tumors identified by an endothelial cell marker CD31. Conversely, an overexpression of HDGF increased vessel formation in melanoma. Therefore HDGF has both mitogenic and proangiogenic activities that renders it a favourable therapeutic target for the treatment of melanoma.

The molecular mechanism underlying HDGF-induced metastasis remains unclear. One possibility is that HDGF facilitates EMT in melanoma, thereby creating a niche environment for metastasis. EMT was originally described by embryologists and occurred in many developmental processes, however it is also a key step in cancer progression when cells acquire invasive and disseminate behaviours [27]. Down-regulation or loss of E-cadherin results in de-differentiation, gain of invasiveness, and promotion of EMT in carcinoma cells including malignant melanoma [28]. Our finding reveals that E-cadherin protein level is down-regulated, whereas vimentin and α-SMA are up-regulated in HDGF-treated melanoma cells. Our recent study demonstrated that HDGF up-regulation is correlated with recurrence, lymph node metastasis and EMT in breast cancer patients [20]. It is likely that HDGF may act by inducting EMT-promoting growth factors, such as transforming growth factor-β (TGFβ) [29]. Because the reciprocal regulation between HDGF and TGFβ exists during liver fibrosis [30], it is plausible that HDGF might induce TGFβ up-regulation and concomitantly promote EMT during melanoma progression. Apart from the facilitation of EMT, our recent study also suggests that HDGF may promote the cytoskeletal remodelling through stimulation of Rho activity and the formation of podosomes that favours the motility of tumor cells [31]. Therefore, HDGF may regulate EMT process and cytoskeletal remodelling for triggering metastasis of melanoma. Future studies are warranted to delineate these hypotheses for the mechanism.

In summary, we presented evidences that HDGF promotes melanoma progression through the activation of tumorigenesis, angiogenesis and metastasis. Therefore, targeting HDGF may well be a novel strategy for the treatment of melanoma and also be used as a diagnostic marker for melanoma.

## Supporting Information

Figure S1 Effects of HDGF on E-cadherin transcriptional activity in B16–F10 melanoma. (A) E-cadherin transcriptional activity of HDGF-treated B16–F10 cells using luciferase assay. (B) Effects of Ad-GFP, Ad-HDGF and Ad-HDGF shRNA on promoter activity of E-cadherin. Data are expressed as mean ± SEM from 3–4 experiments. *P<0.05 compared to Control- (for A with t-test) or Ad-GFP-treated groups (for B with one-way ANOVA with post-doc analysis).
(PDF)
