# Correction: Up-Regulation of Hepatoma-Derived Growth Factor Facilities Tumor Progression in Malignant Melanoma

**DOI:** 10.1371/annotation/02369930-29de-4cd6-a059-9520af0be6b9

**Published:** 2013-06-27

**Authors:** Han-En Tsai, Jian-Ching Wu, Mei-Lang Kung, Li-Feng Liu, Lai-Hsin Kuo, Hsiao-Mei Kuo, San-Cher Chen, Elsa C. Chan, Chieh-Shan Wu, Ming-Hong Tai, Guei-Sheung Liu

One of the words in the article title was spelled incorrectly. The correct title is: "Up-Regulation of Hepatoma-Derived Growth Factor Facilitates Tumor Progression in Malignant Melanoma." 

The correct citation is: Tsai H-E, Wu J-C, Kung M-L, Liu L-F, Kuo L-H, et al. (2013) Up-Regulation of Hepatoma-Derived Growth Factor Facilitates Tumor Progression in Malignant Melanoma. PLoS ONE 8(3): e59345. doi:10.1371/journal.pone.0059345. 

