# Supplementary material for: Up-Regulation of Hepatoma-Derived Growth Factor Facilities Tumor Progression in Malignant Melanoma
Source: PLoS One. 2013 Mar 25;8(3):e59345. doi: 10.1371/journal.pone.0059345 (PMC3607612; doi:10.1371/journal.pone.0059345)
Supplement: Figure S1 — Effects of HDGF on E-cadherin transcriptional activity in B16–F10 melanoma. (A) E-cadherin transcriptional activity of HDGF-treated B16–F10 cells using luciferase assay. (B) Effects of Ad-GFP, Ad-HDGF and Ad-HDGF shRNA on promoter activity of E-cadherin. Data are expressed as mean ± SEM from 3–4 experiments. *P<0.05 compared to Control- (for A with t-test) or Ad-GFP-treated groups (for B with one-way ANOVA with post-doc analysis). (PDF) [file pone.0059345.s001.pdf]

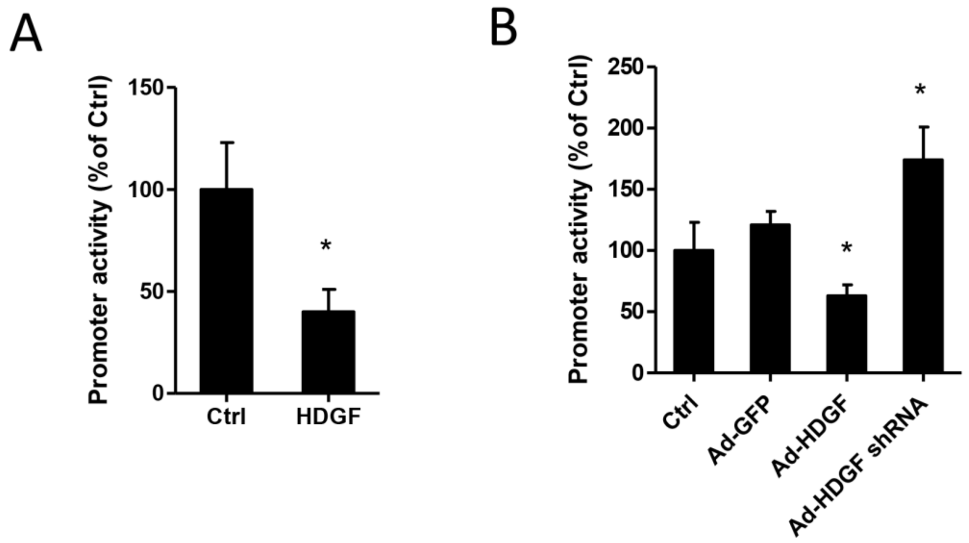

Figure S1. Effects of HDGF on E-cadherin transcriptional activity in B16-F10 melanoma

(A) E-cadherin transcriptional activity of HDGF-treated B16-F10 cells using luciferase assay. (B) Effects of Ad-GFP, Ad-HDGF and Ad-HDGF shRNA on promoter activity of E-cadherin. Data are expressed as mean ± SEM from 3-4 experiments. *$P<0.05$ compared to Control- (for A with t-test) or Ad-GFP-treated groups (for B with one-way ANOVA with post-doc analysis).
